# The Effect of Bigorexia Nervosa on Eating Attitudes and Physical Activity: A Study on University Students

**DOI:** 10.1155/2022/6325860

**Published:** 2022-08-24

**Authors:** Müge Arslan, Nurcan Yabancı Ayhan, Esra Tansu Sarıyer, Hatice Çolak, Ekin Çevik

**Affiliations:** ^1^Üsküdar University, Faculty of Health Science, Department of Nutrition and Dietetics, Istanbul, Turkey; ^2^Ankara University, Faculty of Health Science, Department of Nutrition and Dietetics, Ankara, Turkey

## Abstract

**Purpose:**

This study aims to examine the relationship between physical activity, eating attitude, and bigorexia nervosa among university students.

**Method:**

A questionnaire form consisting of sociodemographic characteristics, eating habits, the Eating Attitude Scale (EAT-40), the International Physical Activity Questionnaire (IPAQ), and the Muscle Appearance Satisfaction Scale (MASS) was conducted on undergraduate students at Üsküdar University.

**Results:**

There were 1006 students who participated in this study with a mean age of 22.58 ± 2.87 years. The median “muscle appearance satisfaction” score of the obese students was statistically higher than the normal weight and underweight students. The median score for “Obesity Anxiety” was statistically higher in underweight students than in obese ones. A one-unit increase in IPAQ scores was related to an elevation of 24.9% in the MASS and a decrease of 17.9% in the EAT-40 scores, while a one-unit increase in MASS scores was related to a reduction of 12.5% in the EAT-40 scores.

**Conclusion:**

Eating attitude is associated with bigorexia nervosa, and as MASS scores increase, EAT-40 scores decrease and as IPAQ scores increase, and EAT-40 scores decrease. In other words, as physical activity increases, muscle strength satisfaction elevates, and in parallel with this increase, a positive eating attitude improves.

## 1. Introduction

The World Health Organization (WHO) defines health as “a state of physical, social and mental well-being and not merely the absence of disease or infirmity.”[1]. The “physical” and “mental” well-being in this definition is closely tied to the body and the body perceptions of individuals. For instance, anxiety about weight gain may be an indication of dissatisfaction with body image. This negative body image arises concerning individuals' bodies and appearance, may cause social appearance anxiety, and may be associated with the deterioration of the “spiritual” well-being in the definition of health [2]. Appearance anxiety is the experience of being evaluated by others according to one's physical appearance, and this can negatively affect individuals in social, professional, and academic life. In addition, various eating disorders that may develop in individuals with social appearance anxiety can also trigger different health problems.

Bigorexia nervosa is a body dysmorphic disorder characterized by individuals' desire to have less fat mass and obsession with increasing muscle mass in this context. It includes environmental factors such as media influence, insecurity, peer influence, and dietary attitudes addition to personality traits such as perfectionism [3, 4]. Evidence in the literature indicates that negative body image and appearance anxiety are becoming common among university students, putting this population at risk for body dysmorphic disorders such as bigorexia [5–7]. In addition, poor eating habits caused by social inability, limited income, and lack of nutritional knowledge in this population are known to be directly related to the increased risk of impaired eating attitudes and nutrition-related noncommunicable diseases that may occur in the future [8, 9].

Eating attitude is a complex phenomenon affected by environmental factors as well as motor, cognitive, social, and emotional development and is described as beliefs, thoughts, feelings, and behaviors towards foods [10, 11]. A recent meta-analysis reported a statistically significant relationship between the drive for muscularity, muscle dysmorphia, and impaired eating attitudes [12]. Another study found that muscle dysmorphia and anorexia nervosa show common symptomatic similarities such as impaired body image, disordered eating, and increased exercise behavior [13]. In this regard, it is of great importance to determine the eating attitudes and to develop the necessary policies for health promotion of the young population, which can be seen as the target group to reduce the prevalence of NCDs and to “achieve the goal of healthy individuals and a healthy society.” This study aims to examine the relationship between physical activity, eating attitude, and bigorexia nervosa in university students.

## 2. Method

The population of this cross-sectional and descriptive study consists of undergraduate university students enrolled in Üsküdar University in the 2021-2022 academic year. The sample of the study was obtained by choosing a simple random sample, while the sample size was calculated using the sample formula with a certain universe, and it was calculated that 400 university students would be sufficient for the study to be carried out based on a sample size calculation made with sampling error of 0.05 and 95% confidence. The study was begun after the necessary permission was received from the Üsküdar University Non-Interventional Research Ethics Committee (numbered 61351342/January 2022-10). A questionnaire form consisting of demographic characteristics, questions about disease status and eating habits (water consumption, skipping meals, etc.), the Eating Attitudes Test-40 (EAT-40), the Muscle Appearance Satisfaction Scale (MASS), and the International Physical Activity Questionnaire (IPAQ) was conducted face-to-face to 1006 voluntary participants after consent forms were signed.

### 2.1. Body Mass Index

Body mass index (BMI) is used frequently in the obesity classification of the WHO for the evaluation of obesity in adults. BMI values are obtained by dividing the individual's body weight (kg) by the square of the height (m^2^). According to the WHO, BMI value below 18.5 kg/m^2^ signifies that the individual is underweight, while BMI value between 18.5 and 24.99 kg/m^2^ is defined as normal weight, between 25.0 and 29.9 kg/m^2^ as overweight, and over 30 kg/m^2^ defined as obesity [14].

### 2.2. Eating Attitude Scale (EAT-40)

The EAT-40 was developed by Garner and Garfinkel [15] to determine the presence of eating disorder attitudes in individuals, and this scale was subsequently adapted into Turkish by Savaşır and Erol [16]. It is a 6-point (always, very often, often, sometimes, rarely, and never) Likert-type self-report scale consisting of 40 items. The final score is determined by summing the scores given by the respondent to each question [15, 16]. Those with a total EAT-40 score of 30 or higher are accepted as having “disrupted eating,” while those with scores of under 30 are considered to practice “normal eating” [16].

### 2.3. Muscle Appearance Satisfaction Scale (MASS)

The survey was developed by Mayville et al. [17] to evaluate cognitive, sensory, and behavioral dimensions of muscle dysmorphia. It is a 5-point Likert-type scale (1: strongly disagree; 5: agree) consisting of 19 items in total based on self-report. The final score is determined by summing up the answers given to each question by the participants. Items 1, 4, and 14 on the scale are reverse coded. Turkish validity and reliability studies of the scale were carried out by Selvi and Bozo [18].

### 2.4. International Physical Activity Questionnaire (IPAQ)

The IPAQ was developed by the researchers from various countries with the support of the WHO and the Centers for Disease Control and Prevention (CDC). Turkish validity and reliability studies were done by Öztürk [19]. It provides information about the time individuals have spent walking, moderate-to-vigorous, and vigorous activities in the last seven days. The time spent sitting is considered a separate question. Calculation of the total score of the short form includes the sum of the duration (minutes) and frequency (days) of walking, moderate-intensity activity, and vigorous activity. It allows the calculation of metabolic equivalent (MET) by measuring frequency, duration, and physical activity intensity level and presents the amount of physical activity per week. After calculating the weekly working hours (MET-hours/week), the physical activity group is determined according to physical activity intensity [19].

### 2.5. Statistical Analysis

Descriptive statistics for categorical variables (demographic characteristics) are presented as frequency and percentage. The conformity of the numerical variables to the normal distribution was checked with the Shapiro-Wilk Test. Median (min-max) values are given for data where the descriptive statistics of numerical variables do not show normal distribution. The Mann-Whitney *U* test was used in the comparison of two independent groups that did not show normal distribution, and the Kruskal-Wallis H test was used in the comparison of more than two groups. The results of multiple comparison tests are expressed in tables as letters next to medians. Relationships between the scales were determined by Spearman rank-differences correlation coefficients. The values used in the interpretation of the correlation coefficients were <0.2, 0.2–0.4, 0.4–0.6, 0.6–0.8, and >0.8 corresponding to very weak, weak, moderate, high, and very high correlations, respectively [20]. Regression analysis was used to test effects between variables. Regression analysis provides an explanation of the relationship between two related variables, namely a dependent variable and an independent variable, with mathematical equivalence [21]. In all calculations and interpretations, statistical significance was considered at *p* < 0.05, *p* < 0.01, and *p* < 0.001, and bidirectional hypotheses were established. Statistical analysis of the data was performed with IBM SPSS Statistics 26 (IBM Corp., Armonk, NY, USA).

## 3. Results

The average age of students was found as 22.58 ± 2.87 years and 28.2% of the participants were male and 71.8% were female. Regarding BMI, most students' weights were in the normal range, and the frequencies for normal BMI were 81.2% for all students, 59.5% for males, and 89.8% for female students. The frequency of students consuming 2–2.5 L/d of water was 33.4%. The rate for skipping of meal was 47.8%, and the most frequently skipped meal was breakfast (42.7%) (Table 1).

The median MASS score of male students [55 (21–88)] was higher than that of females [46.5 (19–90)] (*U* = 76786; *p* < 0.001). The participants' median MASS scores studying in other departments [55 (21–88)] were higher compared to students in health science departments [47 (19–90)] (*U* = 88987.5; *p* < 0.001). Students consuming more than 2.5 L/d of water [57 (27–88)] and 2–2.5 L/d of water [54 (22–89)] had higher MASS median score than students consuming less than 1.5 L/d of water [34 (19–85)] (*H* = 140.403; *p* < 0.001). Finally, the MASS median score of students who skipped meals [51 (19–89)] was higher compared to those who did not skip meals [49 (21–90)] (*U* = 111207.5; *p* < 0.01). Students who skipped night meals had a statistically higher MASS median score [57.5 (28–74)] than the students who skipped lunch [41 (19–85)] (*H* = 21.919; *p* < 0.01).

The median EAT-40 score of students consuming less than 1.5 L/d of water [168 (41–240)] was higher compared to the students consuming more than 2.5 L/d of water [150.5 (58–240)] (*H* = 33.336; *p* < 0.001), and students who skipped meals [165 (41–240)] were found to be statistically higher EAT-40 score than students who did not skip meals [159 (71–240)] (*U* = 97293.5; *p* < 0.001) (Table 2).

When the median scores for subscales of MASS were evaluated, it was found to be statistically higher “Bodybuilding Dependence” median score for male students [14 (5–24)] compared to female students [11 (5–25)] (*U* = 78742; *p* < 0.001); “Muscle Checking” median score for males [11 (4–19)] compared to females [8 (4–20)] (*U* = 78788; *p* < 0.001); “Substance Use” median score of male students [11 (4–20)] compared to female students [8 (4–20)] (*U* = 78036.5; *p* < 0.001); “Injury” median score of male students [8 (3–15)] compared to female students [7 (3–15)] (*U* = 76993.5; *p* < 0.001); and “Muscle Satisfaction” median score of males [10 (3–15)] compared to female students [9 (3–15)] (*U* = 90918.5; *p* < 0.01).

According to the EAT-40 subscales, median score of “Obesity Anxiety” for male [16.5 (4–24)] was found to be statistically higher than for female [16 (4–24)] (*U* = 88760.5; *p* < 0.01) (Table 3).

It was found that the median score of the obese students [12 (8–15)] in the “Muscle Satisfaction” subscale was statistically higher compared to normal weight [10 (3–15)] and underweight students [10 (3–15)] (*H* = 20.996; *p* < 0.001), and the median score for “Obesity Anxiety” subscale of underweight students [17 (4–24)] was found to be statistically higher than obese students [15 (10–22)] (*H* = 8.071; *p* < 0.05) (Table 4).

The correlation between the IPAQ, MASS, and EAT-40 scores of the university students participating in the study is given in Table 5. A positive, statistically significant but weak correlation was found between IPAQ and MASS scores (*s* = 0.249; *p* < 0.001 ). On the other hand, a statistically significant, but very weak correlation was found between IPAQ and EAT-40 scores (*s* = −0.179; *p* < 0.001 ). Likewise, there was a statistically significant but very weak correlation between MASS and EAT-40 scores (*s* = −0.125; *p* < 0.001 ). Finally, when the IPAQ score increased, it was found to be a 24.9% increase in the MASS score and a 17.9% decrease in the EAT-40 score. When the MASS score increased, EAT-40 score decreased by 12.5%.

The relationship between IPAQ, EAT-40, and MASS score is given in Table 6. It was found that the IPAQ score is significantly related to the MASS score. Accordingly, IPAQ scores (*β* = 0.001; *t* = 2.965; *p* < 0.01) explained 0.9% of the variance of MASS scores (*R*^2^ = 0.093; *F* = 8.790; *p* < 0.01). In addition, MASS score was found to be significantly related to EAT-40 score (*β* = -0.050; *t* = −3.220; *p* < 0.01), and it was found that EAT-40 scores explained 0.9% of the variance of MASS scores (*R*^2^ = 0.101; *F* = 10.368; *p* < 0.01).

## 4. Discussion

It is essential to evaluate and improve eating attitudes and physical activity in the young population in order to achieve the “healthy society” goal. In this regard, our study aims to examine the eating attitudes of university students and to evaluate their relationship with bigorexia nervosa.

Most of the participants in this study had normal BMI in terms of WHO classification, and the percentage of female students classified as normal weight were found more in number than male students. In a comprehensive research conducted by Peltzer et al. [22], the body weights of 15746 undergraduate students in 22 countries were evaluated. Similarly, the average BMI of undergraduate students in all countries participating in the study was in the normal range of 22.5 kg/m^2^, and the prevalence of obesity was higher in male students compared to female students. Another recent study on medical students stated higher overweight and obesity rates in males than females [23]. This can be explained by the fact that the number of male students participating in our study is less than females and the percentage of male students receiving education in the field of health is lower than females. The reason that female students with normal body weight are higher than male students can be explained by the fact that female students pay more attention to their nutrition to stay slim due to their fear of becoming fat and their desire to be liked more than male students.

In our study, the majority of students consumed 2–2.5 L/d of water at recommended levels. A recent study with university students in Czechia and Slovakia reported that water consumption in this population was over 1.5 L/d [24]; a different study reported that the total fluid intake among university students exceeded 1.5 lt/day, but there was not enough water consumption [25]. These differences in study results may be due to individual differences, or they may not be based on food consumption records.

In this study, it was found that almost half of the students skipped their meals and the most skipped meal was found to be breakfast. According to a meta-analysis of 35 studies, it was observed that the frequency of meal skipping among young adults ranged between 5 and 83%, and the most frequently skipped meal was breakfast [26]. Another recent meta-analysis focused on the breakfast skipping habits in a total of 21,958 university students from 28 countries. Accordingly, the frequency of skipping breakfast was found to be 48% among university students [27]. Contrary to these data, 73% of university students participating in another study conducted in Turkey reported that they regularly consume breakfast [28]. The reasons why university students skip breakfast may be that they live far from their family home, they care more about the time allocated to sleep, and their urge to consume as few meals as possible due to economic concerns. When the IPAQ results, which give information about the daily physical activities and physical activity levels of the individuals, are evaluated, although the median IPAQ score of male students is partially higher than females, this difference was not statistically significant and the physical activity status of our study population was evaluated as “inactive.” In a recent cohort study covering the pre-and post-university period, it was stated that the frequency of physical activity among university students decreased with the start of university, and the frequency of exercise was higher among males [29]. Two different studies have shown that, contrary to our data, the level of physical activity among university students is sufficient. A systematic review of 21 studies in total reported that the level of physical activity among university students was moderate [30]. Another study found that half of the university students participating in the study were less active/inactive [31]. On the other hand, the data obtained from these studies indicate that especially males are more active or, similar to our study, although the level of physical activity is higher in males, it is not statistically significant [32, 33]. The prevalence of low physical activity among the students participating in our study may be the effect of an ongoing sedentary period due to the pandemic. In our study, there was no statistically significant relationship between the department of education and IPAQ scores. In one of the studies examining the physical activity status of different undergraduate departments, it was stated that the frequency of adequate physical activity was higher among medical students [33]. In our study, IPAQ scores do not differ according to BMI. Studies evaluating physical activity status and BMI are based on the fact that BMI is an indicator of body fat percentage. Accordingly, while the relationship between physical activity and BMI in nonobese individuals is weak; it is highly associated with obese individuals [34]. A large cohort study noted that among people with the same BMI, those who were more active had a lower body fat percentage [35]. The fact that this relationship was not found in our study may be since most of the students participating in our study were within the normal weight limits.

Studies investigating the motivation of university students to workout have revealed that appearance anxiety and the desire to be healthy were the most important reasons pushing this population to be physically active [29, 36]. Likewise, in our study, it was observed that as the physical activity levels of the students increased, their muscle strength satisfaction also increased. In a study conducted with adolescents, an increase in sedentary lifestyle among students participating in the study was associated with a decrease in body image scores [37]. In another study on Norwegian university students indicated that female students studying sports science had higher body appreciation scores compared to other departments [38]. In a recent review of 210 publications, it has been reported that positive body image is associated with greater participation in physical activity and sports [39]. In addition, studies show that body image perception is not only linked to physical activity but also to eating behaviors [40, 41]. Indeed, in our study, it was observed that as the physical activity levels of the students increased, their eating attitudes improved. Similarly, a study conducted with university students showed that the eating attitude of individuals who do regular physical activity improves positively [41]. On the other hand, another cross-sectional study on university students revealed that young adults with a high susceptibility to eating disorders have a higher level of physical activity than those with low susceptibility to eating disorders [40]. In the light of all these data, physical activity, muscle strength satisfaction, and the emergence of positive eating attitudes can be associated with the contribution of routine physical activity to positive body image and the desire to protect this improved image by supporting it with diet.

Individuals with signs of muscle dysmorphia may have a normal-looking or rather muscular body but are thought to have a very small, fat, or muscleless body structure. These individuals, in response to appearance concerns, repetitive behaviors, and mental actions, as well as muscularity and weakness become obsessed [42]. In this study, muscle dysmorphia indicators were predominantly associated with male students. Male's bodybuilding addiction, muscle control, and muscle dissatisfaction scores were higher. In the literature, it has been shown that there are more signs of muscle dysmorphia in males [43, 44]. It has been reported to be more common in males who engage in sports that emphasize increased muscle mass or strength gain, especially weightlifting or bodybuilding [42]. One study showed a stronger association between muscle dysmorphia and eating disorder symptoms in males using androgenic steroids. Some of the most common motivations for people who use steroids are to increase muscle mass and strength, improve physical appearance, and relieve symptoms of body dissatisfaction and muscle dysmorphia [45]. However, it has been reported that muscle dysmorphia is also observed in females [44, 46]. Most studies in the literature include male populations without including females. For this reason, it has been shown that both males and females have a high risk of having symptoms of muscle dysmorphia, which may be highly correlated with eating disorders [12].

In our study, no significant difference was found in bodybuilding addiction between normal weight and obese students. While there was no significant difference in bodybuilding addiction and muscle control scores of obese and normal-weight students, muscle dissatisfaction was found to be higher in obese students than in normal weight and underweight students. In a study, a strong positive relationship was reported between body dissatisfaction and obesity in adolescent girls [47]. In another study, it was stated that the children who have a higher perceived body size, it is more likely for them to choose a thinner body size as ideal [48]. It has been observed that high body weight is associated with body image dissatisfaction in adolescent students, and inconsistency and dissatisfaction between muscle tone, breast size, and physical strength and ideal body increase, especially in males [49]. It was found that male university students were not satisfied with both their muscularity and body fat levels, and a higher degree of body dissatisfaction was observed among obese students [50]. An unrealistic desire to increase muscle mass and drastically reduce body fat among obese and trait men may be associated with an increased risk of disordered eating attitudes.

In this study, it was observed that students' eating attitudes did not differ according to gender. Similarly, in a cross-sectional study conducted, although male students were more prone to eating behavior disorders, no significant difference was found between the EAT-40 scores of female students [51]. Similarly, in a study conducted with university students, it was found that there was no statistically significant difference between the eating attitudes of male and female students [52]. Although there was no difference in terms of gender and eating attitude in our study, it was found that the subfactor of the EAT-40 scale, “Obesity Anxiety” score, was statistically higher in male students than in females. Contrary to our study, some studies have found that young women exhibit eating restriction behavior due to bodyweight anxiety [53,54]. In a study conducted by Chen et al. [55], adolescent girls were found to have higher obesity anxiety, while boys were more likely to engage in extreme dietary behaviors such as vomiting, keeping an empty stomach, and avoiding sweets. In addition, bodyweight perception was found to be a more critical factor in triggering eating disorders in boys than in girls. The reason why obesity anxiety was higher in men in our study may be related to high muscle dissatisfaction. Because the point of view focused on muscle increase and decrease in fat ratio, which is more common in men, is associated with deterioration in eating behaviors and body image [56].

In our study, no significant difference was found between the EAT-40 scores of normal weight and obese students. Similar to our results, Sanlier et al. [57] found that there was no significant relationship between students' BMI values and eating attitudes. In the study conducted by Kadıoğlu and Ergün [58] on university students, it was found that the risk of eating disorders was 2 times higher in overweight/obese students compared to normal weight students, and 2.9 times lower in underweight students compared to normal weight students. In addition, in our study, it was found that in the subfactor of the EAT-40 scale the “Obesity Anxiety” scores of underweight students were statistically higher than obese students. Although the data in the literature indicate that a higher risk of disordered eating attitudes is associated with obesity, some studies report an inconsistent relationship between disordered eating attitudes and weight status [59]. This can be explained by the efforts of individuals to reach their ideal body weight, BMI affecting food choice, and related eating attitudes [57].

In this study, there was no significant difference between the EAT-40 scores of health science and other science students. It has been observed that the muscle dysmorphia indicators of the students studying in health-related fields are lower than those in nonhealth fields. In a study, the risk of eating attitude disorder was found to be higher in students studying in the field of sports than in students receiving nutrition education [60]. In another study, it was reported that the food practices of students who received nutrition education were affected, but this was not enough to obtain a healthy diet and reduce body dissatisfaction [61]. A study by Tavolacci et al. [8] reveals that there was no significant difference in terms of eating disorder risk between students studying both in the field of health and in other fields. This situation can be explained by the fact that students studying health, perceive health both physically and spiritually, and they prefer their eating habits based on healthy nutrition rather on body types. Students studying sports have a higher chance of developing an eating attitude disorder; this may be explained by the fact that students studying sports have a strong desire to have a muscular body structure and, as a result, their eating attitudes are solely in this direction and unbalanced.

In our study, it was found that the median EAT-40 score and muscle dysmorphia indicators of the students who skipped meals were statistically higher than the students who did not skip meals. While muscle dysmorphia indicators were observed to be higher in those who skipped meals at night; there was no difference between the skipped meal and the EAT-40 scores. In the studies conducted with university students, it was observed that students who skip meals tend to have a higher risk of eating disorders [60, 62]. There is also a study reporting that there is no significant difference between skipping meals and eating disorders [63]. Another study found that fasting, skipping meals, vomiting, and laxative use were common in adolescents with low body satisfaction and unhealthy weight control behaviors [64]. The habit of skipping meals can be explained by the fact that students who do not have body satisfaction and tend to eat disorders skip meals to achieve the desired body image and maintain weight, and they want to starve themselves.

## 5. Conclusion

This study suggested that as students' physical activity levels increased, so did their satisfaction with their muscle strength and their eating attitudes. On the other hand, as eating attitudes improve, muscle satisfaction may increase as a measure of body image. These findings are beneficial in creating health and education programs that can support university students develop a positive body image and encouraging healthy eating habits. First, although the EAT-40 scale was developed based on attitudes, feelings, and behaviors related to eating, it is mostly used to define the risk of eating disorders, and it may not be sufficient for the accurate evaluation of general eating attitudes or specifically bigorexia nervosa. Second, the subjective nature of the EAT-40, the MASS, and the IPAQ inventories used in this study may have created a bias in the data shaped by participant statements. In this regard, comprehensive studies supported by objective measurements on the effects of physical activity, eating attitudes, and other factors on body image in university students are needed.

## Figures and Tables

**Table 1:** Descriptive statistics of demographic and nutritional findings of university students.

BMI classification	Male		Female		Total	
	*n*	%	*n*	%	*n*	%
Underweight	3	1.1	36	5.0	39	3.9

Normal	169	59.5	648	89.8	817	81.2

Overweight	100	35.2	35	4.8	135	13.4

Obese	12	4.2	3	0.4	15	1.5

Age ($\bar{x}\pm s$)	23.31 ± 2.49		22.29 ± 2.95		22.58 ± 2.87	

Department						
Health sciences	155	54.6	545	75.5	700	69.6
Other sciences	129	45.4	177	24.5	306	30.4

Daily water consumption						
0–1.5 L/d	33	11.6	120	16.6	153	15.2
1.5 < 2 L/d	96	33.8	199	27.6	295	29.3
2 < 2.5 L/d	93	32.7	243	33.7	336	33.4
>2.5 L/d	62	21.8	160	22.2	222	22.1

Skipping meal						
Yes	186	65.5	295	40.9	481	47.8
No	98	34.5	427	59.1	525	52.2

Skipped meal						
Breakfast	90	48.4	115	39.1	205	42.7
Lunch	53	28.5	77	26.2	130	27.1
Dinner	5	2.7	18	6.1	23	4.8
Mid-morning	17	9.1	48	16.3	65	13.5
Afternoon	8	4.3	15	5.1	23	4.8
Night	13	7.0	21	7.1	34	7.1

The median IPAQ scores of the students consuming 2.5 L/d of water and more [657 (0–10179)] was higher that students consuming 1.5–2 L/d [337.5 (0–10533)] and 0–1.5 L/d [273 (0–8127)] of water (*H* = 83.166; *p* < 0.001). The median IPAQ score of students who skipped meals at night [764.5 (0–3450)] was statistically higher compared to those who skipped breakfast [372 (0–10533)] (*H* = 21.893; *p* < 0.01).

**Table 2:** Comparison of demographics and nutritional findings of university students with IPAQ, MASS, and EAT-40 scores.

Gender	IPAQ	MASS	EAT-40
	Median (min-max)	Median (min-max)	Median (min-max)
Male	522.5 (0–10533)	55 (21–88)	155 (41–240)
Female	502.5 (0–9763.5)	46.5 (19–90)	162 (59–240)

U	97927	76786	96815.5
*p*	0.267	<0.001^*∗∗∗*^	0.169

BMI classification			
Normal	480 (0–4566)	41 (19–90)	167 (41–223)
Underweight	514.5 (0–10533)	49 (19–88)	161 (59–240)
Overweight	495 (0–10179)	51 (22–79)	155 (58–240)
Obese	106.5 (0–8316)	44 (31–88)	160 (80–196)

H	3.368	3.004	5.390

*p*	0.338	0.391	0.145
Department			
Health sciences	504 (0–10179)	47 (19–90)	161 (58–240)
Other sciences	494.5 (0–10533)	55 (21–88)	163 (41–240)

U	105602.5	88987.5	101176.5
*p*	0.723	<0.001^*∗∗∗*^	0.162

Daily water consumption			
0–1,5 L/d	273^a^ (0–8127)	34^a^ (19–85)	168^b^ (41–240)
1,5–2 L/d	337.5^a^ (0–10533)	39^ab^ (19–90)	163^ab^ (71–240)
2–2,5 L/d	568.5^ab^ (0–8316)	54^b^ (22–89)	158.5^ab^ (72–235)
>2,5 L/d	657^b^ (0–10179)	57^b^ (27–88)	150.5^a^ (58–240)

H	83.166	140.403	33.336
*p*	<0.001	<0.001	<0.001

Skipping meal			
Yes	465 (0–10533)	51 (19–89)	165 (41–240)
No	537 (0–9763.5)	49 (21–90)	159 (71–240)

U	119519.5	111207.5	97293.5
*p*	0.142	0.001	<0.001

Skipped meal			
Breakfast	372^a^ (0–10533)	53^b^ (19–84)	160 (41–240)
Lunch	397.5^ab^ (0–8265)	41^a^ (19–85)	177 (59–240)
Dinner	567.3^b^ (0–1257)	56^bc^ (40–80)	163 (98–235)
Mid-morning	583^b^ (0–1191)	45^ab^ (24–89)	168 (72–235)
Afternoon	393^ab^ (0–3154.5)	57^bc^ (23–71)	171 (122–218)
Night	764.5^c^ (0–3450)	57.5^c^ (28–74)	170.5 (80–223)

H	21.893	21.919	8.424
*p*	0.001	0.001	0.134

IPAQ: International Physical Activity Scale, MASS: Muscle Appearance Satisfaction Scale, EAT-40: Eating Attitude Test-40., U: Mann-Whitney *U* Test; *H: Kruskal-Wallis H Test.* The difference between medians that do not have a common letter is significant (*p* < 0.05).

**Table 3:** Comparison of MASS and EAT-40 subfactor scores of university students by gender.

MASS	Gender	Median (min-max)	U	*p*
Bodybuilding depedence	Male	14 (5–24)	**78742**	**<0.001**
	Female	11 (5–25)		
Muscle checking	Male	11 (4–19)	**78788**	**<0.001**
	Female	8 (4–20)		
Substance use	Male	11 (4–20)	**78036.5**	**<0.001**
	Female	8 (4–20)		
Injury	Male	8 (3–15)	**76993.5**	**<0.001**
	Female	7 (3–15)		
Muscle satisfaction	Male	10 (3–15)	**90918.5**	**0.005**
	Female	9 (3–15)		

*EAT-40*				
Obesity anxiety	Male	16.5 (4–24)	**88760.5**	**0.001**
	Female	16 (4–24)		
Dieting behaviour	Male	28 (7–42)	99342	0.442
	Female	28 (8–42)		
Social pressure	Male	12 (3–18)	97698.5	0.242
	Female	12 (3–18)		
Preoccupation for weakness	Male	12 (3–18)	99261.5	0.429
	Female	12 (3–18)		

MASS: Muscle Appearance Satisfaction Scale, EAT-40: Eating Attitude Test-40. U: Mann-Whitney *U* Test; *H: Kruskal-Wallis H Test*.

**Table 4:** Comparison of MASS and EAT-40 subfactor scores of university students according to BMI Classification.

MASS	BMI classification	Median (min-max)	H	*p*
Bodybuilding depedence	Underweight	9 (5–25)	4.526	0.210
	Normal	12 (5–25)		
	Overweight	12 (5–23)		
	Obese	10 (5–24)		

Muscle checking	Underweight	6 (4–20)	5.091	0.165
	Normal	9 (4–20)		
	Overweight	10 (4–19)		
	Obese	10 (5–18)		

Substance use	Underweight	8 (4–20)	1.351	0.717
	Normal	9 (4–20)		
	Overweight	9 (4–20)		
	Obese	8 (4–18)		

Injury	Underweight	7 (3–15)	1.636	0.651
	Normal	7 (3–15)		
	Overweight	7 (3–15)		
	Obese	6 (3–15)		

Muscle satisfaction	Underweight	10^a^ (3–15)	20.996	<0.001
	Normal	10^a^ (3–15)		
	Overweight	11^ab^ (3–15)		
	Obese	12^b^ (8–15)		

*EAT-40*				

Obesity anxiety	Underweight	17^b^ (4–24)	8.071	0.045
	Normal	16^ab^ (4–24)		
	Overweight	16^ab^ (4–24)		
	Obese	15^a^ (10–22)		

Dieting behaviour	Underweight	30 (7–42)	6.737	0.081
	Normal	28 (8–42)		
	Overweight	27 (7–42)		
	Obese	28 (14–38)		

Social pressure	Underweight	12 (3–18)	2.303	0.512
	Normal	12 (3–18)		
	Overweight	12 (4–18)		
	Obese	13 (8–17)		

Preoccupation for weakness	Underweight	13 (3–18)	6.861	0.076
	Normal	12 (3–18)		
	Overweight	12 (3–18)		
	Obese	12 (7–17)		

MASS: Muscle Appearance Satisfaction Scale, EAT-40: Eating Attitude Test-40. U: Mann-Whitney *U* Test; *H: Kruskal-Wallis H Test.* The difference between medians that do not have a common letter is significant (*p* < 0.05).

**Table 5:** Correlation coefficients between IPAQ, MASS, and EAT-40 scores.

		IPAQ	MASS
MASS	s	0.249	1.000
	p	<0.001	.

EAT-40	s	−0.179	−0.125
	p	<0.001	<0.001

IPAQ: International Physical Activity Scale, CGMD: Muscle Appearance Satisfaction Scale, EAT-40: Eating Attitude Test-40. s: Spearman's Rank Differences Correlation.

**Table 6:** Regression analysis on the effect of IPAQ and EAT-40 scores on MASS scores.

	Model	*β*	Std. Error	*t*	*p*	*F*	*p*
MASS	(Constant)	47.199	0.616	76.569	<0.001	8.790	0.003
	IPAQ	0.001	0.000	2.965	0.003		
*R* = 0.093; *R*^2^ = %0.9; adjusted *R*^2^ = %0.8							

MASS	(Constant)	55.880	2.444	22.865	<0.001	10.368	0.001
	EAT-40	−0.050	0.015	−3.220	0.001		
*R* = 0.101; *R*^2^ = %1; adjusted *R*^2^ = %0.9							

IPAQ: International Physical Activity Scale, MASS: Muscle Appearance Satisfaction Scale, EAT-40: Eating Attitude Test-40. s: Spearman's Rank Differences Correlation.

## Data Availability

The data used to support the findings of this study are available from the corresponding author upon request.
